# Surprise on Popping the Popliteal Swelling: A Case Report

**DOI:** 10.5704/MOJ.2207.015

**Published:** 2022-07

**Authors:** JS Chong, A Shukriah, CR Mohd-Atiq, J Raeross

**Affiliations:** 1 Department of Orthopaedics, Hospital Tengku Ampuan Afzan, Kuantan, Malaysia; 2 Department of Orthopaedics, Sultanah Nur Zahirah Hospital, Kuala Terengganu, Malaysia; 3 Department of Orthopaedics, Hospital Melaka, Melaka, Malaysia; 4 Department of Orthopaedics, Hospital Bintulu, Bintulu, Malaysia

**Keywords:** popliteal, swelling, popliteal artery aneurysm, Baker’s cyst

## Abstract

Popliteal swelling is a common complaint seen in the practice of orthopaedics. Although imaging is useful to aid in the diagnosis of popliteal swelling pre-operatively, definitive diagnosis is often obtained post-operatively through histopathological report of the swelling. Baker’s cyst arises medially and hence usually spares the posterolaterally located neurovascular bundle until it becomes larger in size. A thrombosed aneurysm can mimic that of Baker’s cyst on computed tomography (CT) imaging in view of its location and the absence of contrast within the lesion. Diagnosis of a popliteal swelling with neural or vascular compression is not as straightforward and surgeons should be well aware that intra-operative findings may differ from diagnosis made pre-operatively. Meticulous exploration is pertinent in identifying the origin of the swelling and structures related to it. MRI imaging of the swelling should be done pre-operatively whenever possible.

## Introduction

Popliteal swelling is a common complaint seen in the practice of orthopaedics. They are routinely discovered in up to 38% of magnetic resonance imaging scans (MRIs) performed in the symptomatic knee^1^. The developing swelling may cause compression to the popliteal neurovascular bundle with symptoms such as tibial or common peroneal neuropathy, resulting in gastrocnemius muscle atrophy, pseudothrombophlebitic syndrome, and rarely arterial compression with claudication of the lower extremity^2^. Many times, even with a thorough history and examination, it is difficult to ascertain the type of popliteal swelling. Although imaging is useful to aid in the diagnosis of popliteal swelling pre-operatively, definitive diagnosis is often obtained post-operatively through histopathological report of the swelling. Therefore, during the attempt to remove a popliteal swelling, meticulous exploration is pertinent in identifying the origin of the swelling and structures related to it.

In the following, we present an interesting case in our experience of managing a patient with popliteal swelling.

## Case Report

We present the case of a 61-year-old Iban man with underlying polycythemia rubra vera and hypertension. He presented to a general practitioner with right calf pain for a month with claudication distance of 200m, the pain was relieved at rest and reduced when kept at a lower level. He was referred to our hospital for peripheral vascular disease of the right lower limb.

On examination he was generally well with stable vital signs. Local examination of the right lower limb revealed a 4x3cm globular swelling at the popliteal fossa with a well-defined margin, smooth edges, no redness, not warm or tender, firm in consistency, not pulsatile, not reducible and mobile from side to side. Right popliteal pulse was not palpable with reduced pulses and monophasic Doppler over dorsalis pedis artery (DPA) and posterior tibial artery (PTA).

**Fig 1:**
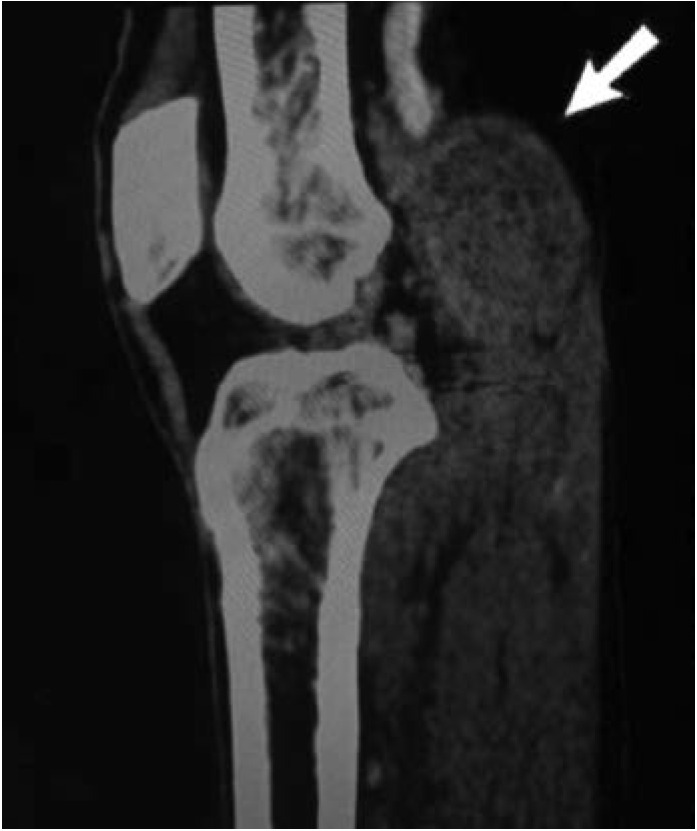
Contrast CT sagittal view showing a non-enhancing mass (arrow) posterior to the knee joint causing obstruction to popliteal artery.

**Fig 2:**
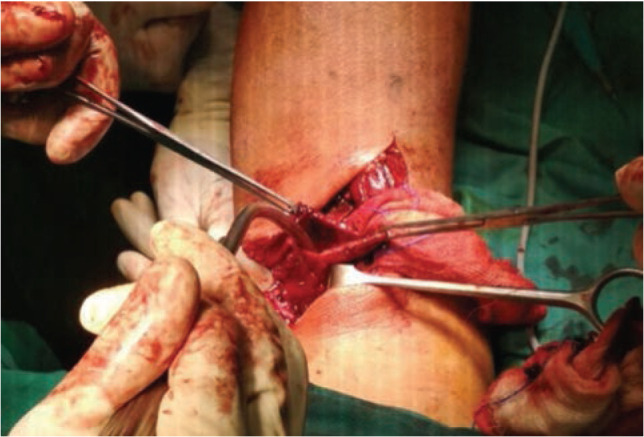
Contrast CT axial view showing a non-enhancing mass (white arrow head) abutting onto a smaller calibre popliteal artery (black arrow).

CT angiography of right knee noted a fairly well-defined cystic mass in the right popliteal fossa region measuring 3.3x3.7x4.0 cm^3^compressing part of the right popliteal artery and vein (in view of its location, possibly a Baker’s cyst) with long segment non-opacification of the popliteal artery from the level of cystic mass within the popliteal fossa until the trifurcation at the level of the proximal tibia with distal reconstitution (Fig. 1 and 2). Patient was diagnosed as right knee Baker’s cyst compressing on popliteal artery complicated with arterial thrombosis. He was then electively posted for excision biopsy over the right knee a week later.

**Fig 3:**
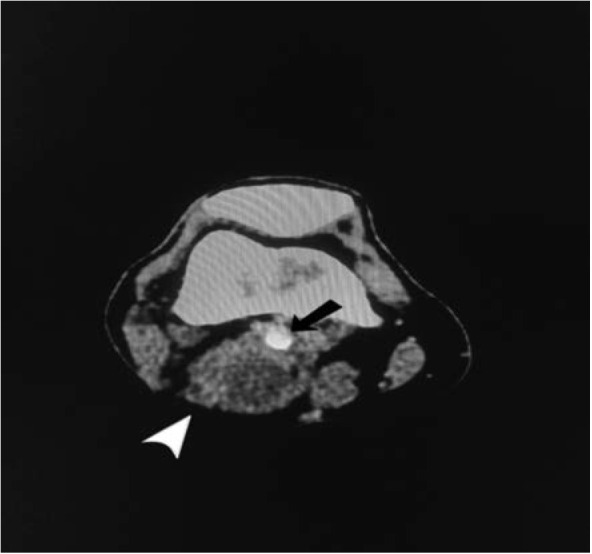
The aneurysmal wall held by Allis forceps on each side. The sac is in direct continuation to the popliteal artery.

Intra-operatively, we discovered a 3x3x2 cm^3^bluish cystic swelling lying over the popliteal artery. The popliteal pulse distal to the swelling was not palpable. The swelling ruptured during manipulation and evacuated blood clots. Upon removal of blood clots, the swelling was found to be in direct communication with the lumen of the popliteal artery. Further exploration noted the swelling was a thrombosed aneursym of the right popliteal artery (Fig. 3). Surgical team was called in. Thrombectomy was done, excess aneursymal wall excised and repaired in layers.

Post-operatively, the right dorsalis pedis and posterior tibial pulses improved, and Doppler assessment was biphasic. Patient was started on heparin infusion and planned for long term oral anticoagulation in view of provoked thrombosis of right popliteal artery secondary to polycythaemia. During his follow-up after three months, patient still complained of claudication pain over the right calf although it was much better compared to his initial presentation. Repeat CT angiogram of the right lower limb showed recurrent thrombosis over the popliteal artery with established collaterals. He was managed conservatively and oral anticoagulation therapy continued.

## Discussion

Baker’s cyst or popliteal synovial cyst is a valvular opening of the posterior capsule, high up on the medial side and deep to the medial head of the gastrocnemius. It is present in up to 40% to 54% of healthy adult knees, based on cadaveric studies^1^. Usually asymptomatic, patients can present with swelling, pain, knee stiffness and compression syndromes. Although Baker’s cyst arises medially and hence usually spares the posterolaterally located neurovascular bundle, occasionally it may develop in size and migrate laterally, leading to neurovascular compression. A study showed that the most common manifestation of neurovascular compression is pseudothrombophlebitis secondary to venous compression (63%) and tibial neuropathy (22%) while isolated arterial compression is the least common phenomenon (7%)^2^. Other differential diagnoses includes ganglion cyst, meniscal cyst, adventitial cyst, synovial sarcoma and artery aneurysm.

Before the advancement of advanced imaging technique such as CT and MRI, combined imaging modalities were often used for the diagnosis of popliteal swelling. Olcott C 4th and Mehigan JT reported a case of Baker’s cyst compressing unto popliteal artery in 1986. Arteriography, arthrography and sonography were employed and initial diagnosis was cystic adventitial disease of the popliteal artery. However, intra-operatively they noticed a Baker’s cyst that was in continuity with the knee joint enclosing a severely inflamed popliteal artery^3^.

In our case, CT angiogram findings of a thrombosed saccular aneurysm mimic that of a Baker’s cyst in view of its location and the absence of contrast within the lesion. C. Chalmeta Verdejo *et al* also reported a similar case as ours where they successfully differentiated Baker’s cysts from popliteal aneurysms with the use of musculoskeletal Doppler ultrasound which could visualise the aneurysm walls and its internal clots^4^. However, the technically demanding nature of musculoskeletal ultrasound limits its use. MRI has been shown as a reliable technique in characterising lesions around the knee and is suggested as the investigation of choice in the pre-operative workup of Baker’s cyst^5^.

Despite thorough pre-operative workup, cautious intra-operative exploration including identification of origin of the swelling is utmost important. Specimen taken should always be sent for histopathological examination. Long term follow-up is needed to monitor recovery of symptoms and to look for recurrence.

In conclusion, diagnosis of a popliteal swelling with neural or vascular compression is not as straightforward as it seems. Surgeons should be aware that intra-operative findings may differ from diagnosis made pre-operatively. CT imaging (with/without contrast) might not be sufficient, and we suggest MRI imaging of the swelling pre-operatively whenever possible, especially in patients with risk of thrombosis such as artherosclerosis, chronic inflammatory disease and hypercoagubility state.

## References

[1] Frush TJ, Noyes FR (2015). Baker’s cyst: Diagnostic and surgical considerations.. Sports Health..

[2] Sanchez JE, Conkling N, Labropoulos N (2011). Compression syndromes of the popliteal neurovascular bundle due to Baker cyst. J Vasc Surg..

[3] Olcott C 4th, Mehigan JT (1986). Popliteal artery stenosis caused by a Baker's cyst.. J Vasc Surg..

[4] Chalmeta Verdejo C, Alegre Sancho JJ, Román Ivorra JA, Ivorra Cortes J (2011). [Popliteal aneurysm simulating a Baker's cyst in a patient with rheumatoid arthritis: a case presentation].. Reumatol Clin..

[5] Marra MD, Crema MD, Chung M, Roemer FW, Hunter DJ, Zaim S (2008). MRI features of cystic lesions around the knee.. Knee..

